# The effect of perceived stress on cognition is mediated by personality and the underlying neural mechanism

**DOI:** 10.1038/s41398-022-01929-7

**Published:** 2022-05-12

**Authors:** Ximei Zhu, Wei Yan, Xiao Lin, Jianyu Que, Yuetong Huang, Haohao Zheng, Lin Liu, Jiahui Deng, Lin Lu, Suhua Chang

**Affiliations:** 1 Peking University Sixth Hospital, Peking University Institute of Mental Health, NHC Key Laboratory of Mental Health (Peking University), National Clinical Research Center for Mental Disorders (Peking University Sixth Hospital), Peking University, Beijing, 100191 China; 2 Research Unit of Diagnosis and Treatment of Mood Cognitive Disorder (2018RU006), Chinese Academy of Medical Sciences, Beijing, 100191 China

**Keywords:** Human behaviour, Pathogenesis, Neuroscience

## Abstract

Perceived stress impairs cognitive function across the adult lifespan, but the extent to which cognition decline is variable across individuals. Individual differences in the stress response are described as personality traits. Substantial individual differences in the magnitude of cognitive impairment that is induced by short-term perceived stress are poorly understood. The present study tested the hypothesis that the relationship between short-term perceived stress and different aspects of cognition is mediated by personality traits. The study included 1066 participants with behavior and neuroimaging data from the Human Connectome Project after excluding individuals with missing variables. In the result, the parallel multiple mediation model demonstrated that the influence of perceived stress on the total and crystalized cognition is mainly mediated by neuroticism (indirect effect = −0.04, *p* < 0.05) and conscientiousness (indirect effect = 0.05, *p* < 0.05) in adults. Cortical thickness value (*n* = 1066) of the right superior frontal gyrus (SFG) showed not only positive correlations with short-term perceived stress and neuroticism, but negative associations with cognition. The chain mediation model found that the right SFG and neuroticism play a small but significant chain mediating effect between stress and total cognition. The strength of the resting-state functional connectivity (*n* = 968) between the left orbitofrontal cortex versus the left superior medial frontal cortex was positively correlated with crystallized cognition and negatively associated with conscientiousness. These results extend previous findings by the impacts of short-term perceived stress on cognitive function is mediated by neuroticism and the right SFG was the underlying neural mechanism.

## Introduction

Perceived stress is a feeling about how much strain individuals perceive when environmental demands exceed their adaptive capacity [1]. The prevalence of perceived stress in adults is 34.8–55%, which is higher than anxiety and depression [2–4]. Perceived stress influences the pathogenesis of physical disease by causing negative affective states, which in turn exert direct effects on biological processes or behavioral patterns that influence disease risk.

Perceived stress or challenge might enhance cognitive performance in some situations while limiting performance in other situations. Mild stress could help to enhance cognitive function, particularly in memory tasks or when the cognitive load is not excessive [5, 6]. Exposure to high, acute stress impairs the complex cognitive function [7, 8]. However, the extent to which cognition can be impaired is often variable across individuals [9]. The extent of perceived stress to cognition decline depends on the individual difference in stress response and brain function [10, 11]. One widely cited explanation derives from the cognitive activation theory of stress agreed that the preference for individual coping styles in response to stress is a characteristic of personality [12, 13]. Personality refers to individual differences in characteristic patterns of thinking, feeling, and behaving [14], which is frequently broken into several traits, including neuroticism, conscientiousness, openness, agreeableness, and extroversion. Evidence suggested that short-term perceived stress exposure affects stability and change of neuroticism [15, 16]. Life events can lead to changes in personality traits and that those different life events may be differently related to specific trait domains [17]. Previous studies indicated that positive life events (enter a new relationship) in last month predict an increase in extraversion [18], and stressful life events in last 3 months predict an increase in neuroticism [19]. Neuroticism mediates the impact of menopause status on depressive symptoms among women [20]. Perceived stress significantly predicts neuroticism, which in turn cumulatively increases depression risk across the life span [21, 22]. Personality trait changes are more strongly related to how individuals perceived in their lives rather than simply the occurrence of such stressful events [23]. Longitudinally, perceiving the event as a negative obstruction was associated with increases in neuroticism, whereas perceiving the event as a positive challenge was associated with increases in extraversion and conscientiousness.

Personality traits also influence the cognitive function across the adult lifespan and correlate to mild cognitive impairment in older adults [24, 25]. Previous studies reported that lower neuroticism and extraversion are associated with better performance on the crystallized and fluid ability tasks [26], while higher openness is associated with higher cognitive performance [27]. Conscientiousness correlates negatively with abstract reasoning, verbal reasoning, as well as fluid cognition [28, 29]. Furthermore, neuroticism and conscientiousness predict performance on the prospective memory tasks [30–32]. Interestingly, personality is more strongly related to crystallized intelligence than to fluid intelligence [33].

Prior imaging studies have observed that stress induces remodeling of the prefrontal cortex (PFC) and affects both the structure and function of PFC [34, 35]. Chronic and acute stress induces cerebral blood flow changes, architectural changes, and dysfunction of the PFC [7, 36]. Perceived stress in healthy adults induces hypoactivation of the working memory-related PFC and the reallocation of neural resources away from executive function networks [37, 38]. Individuals with early life stress exhibit poorer processing speed and working memory, and have smaller volumes of the lateral orbitofrontal cortex and caudate nucleus [39]. According to previous studies, the regional brain structure especially the prefrontal cortex [40, 41], and the functional connectivity have been proved to be related to cognition, as Zhu and colleagues have mentioned that the functional connectivity related to the superior frontal gyrus (SFG) played significant roles in higher-order cognitive functions [42]. A short-term study based on Human Connectome Project (HCP) database also found that personality is associated with the subcortical-medial prefrontal network and the dorsolateral PFC [43]. However, the relationship between stress, personality, and cognition is still unclear, especially in term of neuroimaging. Insights into the role of personality in the stress-cognition relationships are therefore important and may lead to new interventions to target vulnerability to stress-related cognitive decline rather than the manifestations of cognition impairment.

Based on previous studies, we hypothesized that the relationship between short-term perceived stress and different aspects of cognitive function (total, crystallized, and fluid cognition) is mediated by personality traits (neuroticism, conscientiousness, openness, agreeableness, and extroversion) in young adults. The data that were analyzed in the present study were derived from the HCP database, which included behavioral, magnetic resonance imaging (MRI), and genetic data that were collected from 1206 young adults. We sought to form a framework to explain how different personality traits mediate the effect of short-term perceived stress on different aspects of cognition decline and further explore the underlying neuroimaging mechanism by analyzing MRI data to probe the validity of our explanations and posited mechanisms.

## Methods

### Participants

The data in the present study were drawn from the HCP database (March 2017 public data release) from the Washington University-University of Minnesota (WU-Minn HCP) Consortium [44, 45]. The research procedures and ethical guidelines were approved by Washington University’s institutional review board. The WU-Minn HCP Consortium obtained full informed consent from all participants.

### Measurement and statistical analysis

The short-term perceived stress of the participants in the HCP was assessed by the perceived stress scale in the National Institutes of Health (NIH) toolbox [46], which measures the degree of stress a person feels. The scale includes 10 items about levels of experienced stress during the last month. Respondents rate the items on a 5-point Likert with higher scores reflecting higher perceived stress [47]. The total score of the 10 items was used to denote the perceived stress.

Personality was measured by the 60-item version of the Costa and McRae Neuroticism/Extroversion/Openness Five-Factor Inventory (NEO-FFI) [48]. The NEO-FFI is one of the most commonly used questionnaires to capture the major facets of human personality traits, including neuroticism, conscientiousness, openness, agreeableness, and extroversion.

Cognition was measured by a series of tests in the NIH Toolbox cognitive battery [49]. Composite cognition scores from the NIH Toolbox [50] included crystallized cognition composite score (i.e., the ability to solve problems based on prior knowledge and experience, including language decoding and language comprehension) [51], fluid cognition composite score (i.e., the ability to solve novel reasoning problems, which is correlated with executive function, episodic memory, working memory, and processing speed) [51] and the total cognitive function composite score (a combination of both crystallized and fluid scores). Compared with the gold standard composites of cognition (IQ scores of the Wechsler Adult Intelligence Scale), total cognitive function, crystallized cognition, and fluid cognition composite scores showed acceptable internal consistency, excellent test–retest reliability, strong convergent and discriminant validities [52]. The specific description of the NIH Toolbox cognition assessment scale is provided in the Supplementary Methods.

All of the analyses used the following variables as covariates: age, gender, race (categorized as white or other), handedness (Edinburgh Handedness Questionnaire scores), years of education, total household income, body mass index (BMI), adult self-report (ASR) anxious/depressed raw score, alcohol dependence diagnosis (*Diagnostic and Statistical Manual of Mental Disorders*, 4th edition [DSM-IV], criteria for alcohol dependence), tobacco dependence diagnosis (DSM-IV criteria for tobacco dependence), marijuana dependence diagnosis (DSM-IV criteria for marijuana dependence), and positive tests for illicit drugs (including cocaine, tetrahydrocannabinol, opioids, amphetamines, methamphetamine, and oxycontin). The correlations among short-term perceived stress, personality traits, and cognitive function were calculated using partial correlation analyses after controlling the above covariates using SPSS 20 software. False discovery rate (FDR) was used to address the issue of multiple comparisons, and FDR-corrected *p* values (*pval_fdr*) < 0.05 were considered statistically significant.

To explore how personality traits contribute to the association between short-term perceived stress and cognitive function, we used model 4 and 6 in PROCESS [53] to construct simple, parallel multiple, and chain mediation models to analyze whether personality traits (mediation variable) mediate the association between short-term perceived stress (input variable) and cognitive function (output variable). The simple mediation models only explore the mediating effect of a single variable, while the parallel multiple mediation models explore the mediating effect of multiple variables which are parallel without causally influencing each other. If one variable influence another, then chain mediation models would be the appropriate choice. The bootstrap method is used to randomly sample 10,000 times from the original sample to estimate the indirect effect value. If the 95% corrected confidence intervals (CI) of the indirect effect value does not include zero, it suggests that the indirect effect is statistically significant at the 0.05 level [54].

### Magnetic resonance imaging data acquisition and statistical analysis

High-resolution T1-weighted (T1w) and T2-weighted (T2w) structural images were acquired on a 3T Siemens Skyra scanner (Siemens AG, Erlanger, Germany) with a 32-channel head coil at a resolution of 0.7 mm isotropic (field of view [FOV] = 224 × 224, matrix = 320 × 320, 256 sagittal slices, repetition time [TR] of T1w = 2400 ms, TR of T2w = 3200 ms, echo time [TE] of T1w = 2.14 ms, TE of T2w = 565 ms). The structural (T1w and T2w MRI) data used in this study were from the HCP preprocessed pipeline and composed of FreeSurfer summary statistics data, including surface thickness, surface area, and subcortical segmentation volume [55, 56]. Then brain structure data was processed by z transformation to improve normality. Association analysis with the whole-brain structure analysis is a fundamental tool in augmenting understanding of the brain structure related to phenotype data. Linear regression models were constructed to calculate the association between cortical thickness and area with cognition, stress, and personality after controlling the basic covariates above. *pval_fdr* < 0.05 was considered statistically significant. Linear regression models were constructed to calculate the association between cortical and subcortical volume with cognition, stress, and personality after controlling for total intracranial volume and the basic covariates above.

The resting-state functional MRI (rs-fMRI) images were acquired on a 3T Siemens Skyra scanner with a 32-channel head coil at a resolution of 2 mm isotropic (FOV = 208 × 180, matrix = 104 × 90, 72 slices, TR = 720 ms, TE = 33.1 ms). The rs-fMRI data has been preprocessed by the HCP with its uniform method [45, 55, 56]. Only participants with four rs-fMRI data (two scans and two directions for each scan) were used to construct the whole-brain functional network. After preprocessing, the gray matter of the whole brain was parcellated with Shen brain atlas as template [57]. Nodal signals were created by averaging the regional blood oxygen level-dependent signals of all voxels within each region of two directions of two scans. Pearson cross-correlations between all pairwise combinations of region signals were calculated for each participant, followed by *z* transformation to improve normality. Resting-state functional connectivity (FC) between brain areas, which reflects correlations of activity, has broad implications for the interpretation of the brain regions with altered connectivity in the process of cognition and behavior. The linear regression models were constructed between behavior data and the whole-brain functional connectivity (FC) network (250 × 250 regions with 31,125 edges), which reflected correlations between brain regions with altered connectivity and function after controlling the basic covariates [58]. FDR-corrected *p* values for each component using 5000 permutation testing were used to address the issue of multiple corrections, and the component with *pval_fdr* < 0.05 was considered statistically significant. Since the parcellation of the Shen brain atlas is based on objectively functional connectivity analysis [57], we used the Shen brain atlas as the main result. For comparison, we also tried to use the AAL brain atlas as a template, which included 116 brain regions, to repeat the analyses above.

## Results

### Correlation and mediation model for short-term perceived stress, personality, and cognitive function

After deleting individuals with missing variables, a total of 1066 participants with behavior and brain structure data from HCP were used for the analysis. The analysis flowchart is shown in Supplementary Fig. S1. The participants were young adults, 22–37 years old (mean = 28.82 years, standard deviation = 3.67 years), and 492 were male (Table 1). After controlling for covariates, short-term perceived stress negatively correlated with total cognition (*r* = −0.08, *pval_fdr* < 0.05) and fluid cognition (*r* = −0.07, *pval_fdr* < 0.05). Short-term perceived stress negatively correlated with agreeableness and conscientiousness (Table 2). Only neuroticism positively correlated with short-term perceived stress (*r* = 0.41, *pval_fdr* < 0.01). The correlation between openness, extroversion, and short-term perceived stress was nonsignificant. Total cognition positively correlated with openness, and negatively correlated with conscientiousness and neuroticism (Table 2).

**Table 1:** Demographic data of the participants.

Characteristic	*n* (%)
Overall	1066 (100.00)
Gender	
Male	492 (46.20)
Female	574 (53.80)
Age (years), mean (SD)	28.82 (3.68)
Race	
White	799 (75.00)
Other	267 (25.00)
Total household income ($)	
< 39,999	420(39.40)
40,000-99,999	477 (44.70)
≥ 100,000	169 (15.90)
Education (years), mean (SD)	14.93 (1.79)
BMI, mean (SD)	26.42 (5.06)
Adult self-report anxious/depressed problems scale score, mean (SD)	5.81 (5.33)
Substance abuse	
DSM-IV criteria for marijuana dependence	100 (9.40)
DSM-IV criteria for alcohol dependence	60 (5.60)
Positive test for drug	132 (12.40)
DSM-IV criteria for tobacco dependence	221 (20.70)
NIH Toolbox, mean (SD)	
Perceived stress survey	48.40 (9.20)
Total cognition score	112.56 (14.68)
Crystallized cognition score	117.54 (10.02)
Fluid cognition score	115.34 (11.62)
Personality, mean (SD)	
Neuroticism	16.57 (7.37)
Openness	28.35 (6.22)
Conscientiousness	34.44 (5.93)
Agreeableness	33.55 (5.73)
Extroversion	30.68 (6.98)

*n* number, *SD* standard deviation, *DSM-IV* Diagnostic and Statistical Manual of Mental Disorders, 4th edition.

**Table 2:** Correlation between perceived stress, personality traits, and cognition.

	Perceived stress		Total cognition		Crystallized cognition		Fluid cognition	
	Coefficient	pval_fdr	Coefficient	pval_fdr	Coefficient	pval_fdr	Coefficient	pval_fdr
Perceived stress			−0.08	0.02	−0.05	0.14	−0.07	0.04
Neuroticism	0.41	<0.001	−0.09	<0.01	−0.08	0.03	−0.07	0.04
Openness	−0.06	0.07	0.21	<0.001	0.32	<0.001	0.06	0.06
Conscientiousness	−0.18	<0.001	−0.11	<0.001	−0.18	<0.001	−0.04	0.28
Agreeableness	−0.16	<0.001	0.02	0.65	0.07	0.04	−0.02	0.53
Extroversion	−0.04	0.26	−0.06	0.09	−0.11	<0.01	0	0.95

*pval_fdr*, False Discovery Rate (FDR)-corrected *p* value. df = 1052.

**Fig. 1:**
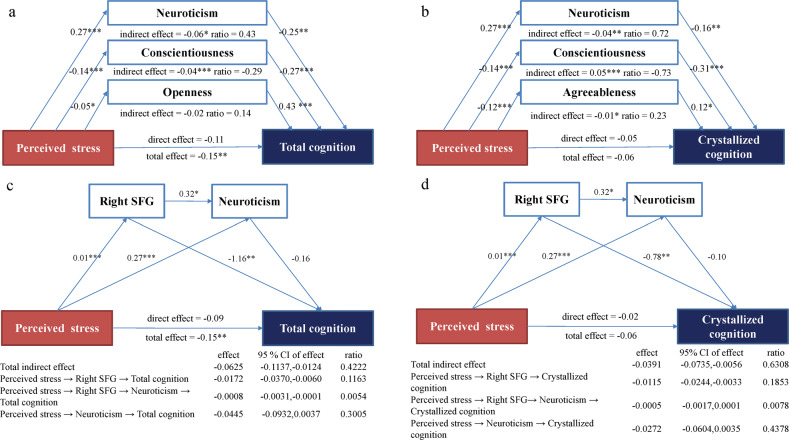
Mediation model of personality and right superior frontal gyrus (SFG) for the relationship between perceived stress and cognition. *p < 0.05, **p < 0.01, ***p < 0.001. CI, confidence intervals. ratio, ratio of indirect effect to the total effect of each mediator. **a** The parallel multiple mediation model of neuroticism and conscientiousness mainly mediated the relationship between perceived stress and total cognition. Openness was nonsignificant in the mediation model. **b** The parallel multiple mediation model of neuroticism, conscientiousness, and agreeableness between perceived stress and crystallized cognition. **c** The chain mediation model of right SFG and neuroticism between perceived stress and total cognition. **d** The chain mediation model of right SFG and neuroticism between perceived stress and crystallized cognition.

The simple mediation analysis found that the relationship between short-term perceived stress and total cognition was mediated by neuroticism, conscientiousness, and openness respectively (Supplementary Table S1). The relationship between short-term perceived stress and crystallized cognition was mediated by neuroticism, conscientiousness, and agreeableness respectively (Supplementary Table S1). No personality trait mediated the relationship between stress and fluid cognition. Personality traits that were significant in the simple mediation analysis were included simultaneously in the parallel multiple mediation model to compare the mediation effect and mediation ratio (i.e., the ratio of the specific indirect effect to the total effect). For total cognition, the mediation effect and ratio of neuroticism (indirect effect = −0.06, ratio = 0.43) was higher than the mediation effect and ratio of conscientiousness (indirect effect = −0.04, ratio = −0.30), whereas the mediation effect of openness was nonsignificant in the parallel multiple mediation model (Fig. 1a). For crystallized cognition, the mediation effect and ratio of neuroticism (indirect effect = −0.04, ratio = 0.72) and conscientiousness (indirect effect = 0.05, ratio = −0.73) were higher than agreeableness (indirect effect = 0.01, ratio = 0.23; Fig. 1b). The mediation effect of neuroticism, openness, and agreeableness had the same direction as the total effect of stress on cognition. In contrast, the mediation effect of conscientiousness was in the opposite direction of the total effect of stress and cognition (Fig. 1). Because of the different mediating directions of personality factors, the total indirect effect was statistically non-significant in the multiple mediation models. The total effect, which was a combination of the direct effect of stress and the indirect effect of personality, indicated that stress was negatively associated with cognition.

### Shared brain structural characteristics between perceived stress, personality, and cognitive function

For the whole-brain analysis after FDR correction, the linear regression models revealed that the cortical thickness of 10 brain regions was significantly associated with short-term perceived stress, including bilateral SFG, bilateral rostral middle frontal gyrus, bilateral caudal middle frontal gyrus, right pars opercularis gyrus, left pars orbitalis gyrus, left pars triangularis gyrus, and left precentral gyrus. Neuroticism was significantly associated with the cortical thickness of right SFG, whereas no significant brain region was associated with the other personality traits (Table 3). Total cognition was associated with cortical areas of 55 brain regions (Supplementary Table S2), and the cortical thickness of 7 brain regions which included bilateral SFG, right rostral middle frontal gyrus, right pars opercularis gyrus, left caudal middle frontal gyrus, left medial orbitofrontal gyrus, and left pars orbitalis gyrus (Table 3). Crystallized cognition was associated with cortical areas of 59 brain regions (Supplementary Table S2) and was associated with the cortical thickness of three brain regions which included right SFG, right rostral middle frontal gyrus, and left pars orbitalis gyrus (Table 3). And fluid cognition was associated with cortical areas of 24 brain regions (Supplementary Table S2). The cortical thickness of six brain regions was positively related to stress and negatively related to total cognition, which included bilateral SFG, right rostral middle frontal gyrus, left caudal middle frontal gyrus, right pars opercularis gyrus, left pars orbitalis gyrus. And the cortical thickness of the right SFG, right rostral middle frontal gyrus, left pars orbitalis gyrus were positively related to stress and negatively related to crystallized cognition (Table 3). Cortical thickness of the right SFG (the only shared brain region among stress, neuroticism, total and crystallized cognition) was positively associated with short-term perceived stress and neuroticism but negatively associated with total cognition and crystallized cognition.

**Table 3:** Cortical thickness of brain regions associated with perceived stress, neuroticism, and cognition in the linear regression models (*n* = 1066).

	Perceived stress			Neuroticism			Total cognition			Crystallized cognition		
	Coefficient (95%CI)	F	pval_fdr	Coefficient (95%CI)	F	pval_fdr	Coefficient (95%CI)	F	pval_fdr	Coefficient (95%CI)	F	pval_fdr
Right superior frontal gyrus	0.75(0.32,1.19)	59.28	0.04	0.51(0.22,0.8)	121.70	0.04	−1.28(−2.06,−0.5)	28.08	0.05	−0.82(−1.33,−0.31)	37.32	0.04
Left superior frontal gyrus	0.63(0.19,1.07)	58.83	0.04	0.44(0.15,0.73)	121.10	0.11	−1.17(−1.96,-0.39)	27.89	0.05	−0.69(−1.2,−0.17)	36.98	0.08
Right rostral middle frontal gyrus	0.67(0.24,1.11)	58.99	0.03	0.24(−0.05,0.53)	119.90	0.43	−1.13(−1.91,−0.35)	27.84	0.05	−0.87(−1.38,−0.36)	37.47	0.03
Left rostral middle frontal gyrus	0.64(0.21,1.08)	58.88	0.04	0.4(0.11,0.69)	120.80	0.15	−1.07(−1.85,−0.29)	27.75	0.06	−0.74(−1.25,−0.24)	37.13	0.07
Right caudal middle frontal gyrus	0.62(0.18,1.06)	58.78	0.04	0.36(0.06,0.65)	120.50	0.20	−1.04(−1.82,−0.25)	27.71	0.07	−0.68(−1.19,−0.17)	36.97	0.08
Left caudal middle frontal gyrus	0.68(0.24,1.12)	58.98	0.03	0.38(0.09,0.68)	120.60	0.15	−1.15(−1.93,−0.36)	27.85	0.05	−0.51(−1.02,0.01)	36.63	0.24
Right pars opercularis gyrus	0.66(0.23,1.1)	58.96	0.03	0.38(0.09,0.67)	120.60	0.15	−0.21(−0.99,0.56)	27.05	0.05	−0.46(−0.97,0.05)	36.57	0.28
Left pars orbitalis gyrus	0.63(0.19,1.06)	58.83	0.04	0.16(−0.13,0.45)	119.60	0.63	−1.19(−1.96,−0.42)	27.95	0.05	−0.87(−1.37,−0.36)	37.48	0.03
Left pars triangularis gyrus	0.68(0.24,1.11)	58.98	0.03	0.29(−0.01,0.58)	120.10	0.36	−0.83(−1.61,−0.04)	27.45	0.19	−0.57(−1.08,−0.05)	36.74	0.17
Left medial orbitofrontal gyrus	−0.03(−0.46,0.4)	57.77	0.90	0.10(−0.19,0.39)	119.50	0.82	−1.10(−1.87,−0.33)	27.83	0.05	−0.62(−1.12,−0.12)	36.87	0.10
Left precentral gyrus	0.67(0.24,1.1)	58.99	0.03	0.32(0.03,0.61)	120.30	0.28	−0.21(−0.99,0.56)	27.05	0.79	−0.06(−0.56,0.45)	36.22	0.92

*pval_fdr*, False Discovery Rate (FDR)-corrected *p* value. df = 1052.

To further analyze the role of right SFG, we constructed the chain mediation model of the right SFG and neuroticism between short-term perceived stress and total/crystallized cognition (Fig. 1c, d). For total cognition, the sequential chain mediation effect of the right SFG and neuroticism between stress and total cognition was small but significant (effect = −0.0008, ratio = 0.0054), and the mediation effect of the right SFG between stress and total cognition was also small and significant (effect = −0.0172, ratio = 0.1163). For crystallized cognition, the sequential chain mediation effect of the right SFG and neuroticism between stress and crystallized cognition were insignificant, whereas the mediation effect of the right SFG between stress and crystallized cognition was significant (effect = −0.0115, ratio = 0.1853). The relationship between stress and total/crystallized cognition was mediated by the right SFG, and the relationship between stress and total cognition was slightly mediated by the sequential chain mediation effect of the right SFG and neuroticism.

### Functional connectivity associated with conscientiousness and crystallized cognition

**Fig. 2:**
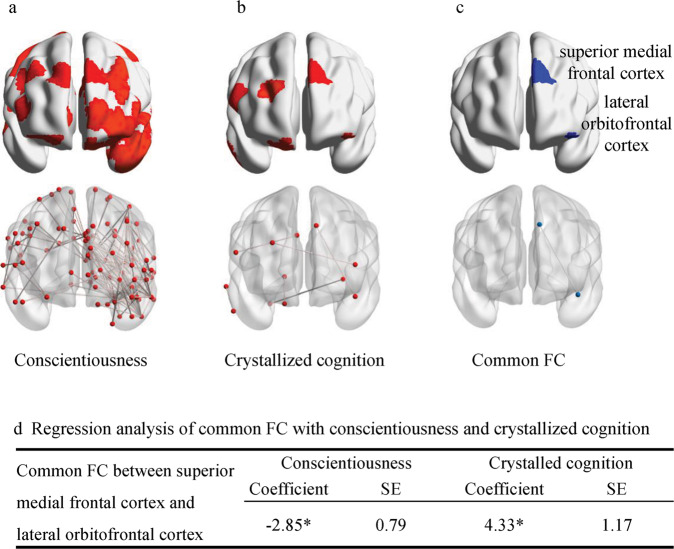
Functional connectivity (FC) associated with conscientiousness and crystallized cognition using the Shen atlas (*n* = 968). *pval_fdr < 0.05. SE, standard error. **a** Activation area and functional connectivity of conscientiousness included 142 edges and 100 nodes. **b** Activation area and functional connectivity of crystallized cognition included six edges and twelve nodes. **c** Functional connectivity of the left lateral orbitofrontal cortex and left superior medial frontal cortex was the only one shared FC among conscientiousness and crystallized cognition. **d** Linear regression models indicated that activation of FC was negatively related to conscientiousness but positively related to crystallized cognition after controlling for covariates.

After excluding 98 participants without four fMRI scans, the rs-fMRI analysis included 968 participants. The linear regression models showed that all three measures had significant network FC after FDR correction. Further comparisons found overlapping FC in Shen brain atlas between conscientiousness and crystallized cognition, whereas no overlapping FC was found for perceived stress and other personality traits (Fig. S2). Conscientiousness was associated with 142 FCs and 100 nodes, including the bilateral frontal gyrus, bilateral orbitofrontal gyrus, right temporal gyrus, bilateral postcentral gyrus, left occipital gyrus, and bilateral precuneus gyrus (Fig. 2a). Crystallized cognition was associated with 6 FCs and 12 nodes, including the bilateral SFG, right temporal gyrus, bilateral orbitofrontal cortex, right precentral gyrus, right cingulate gyrus, right lingual gyrus, and right fusiform gyrus (Fig. 2b). Functional connectivity between the left lateral orbitofrontal cortex (OFC) and left superior medial frontal cortex was positively related to crystallized cognition and negatively related to conscientiousness after controlling for covariates (Fig. 2c, d). However, comparisons of results did not find overlapping FC in the AAL atlas (Fig. S3).

## Discussion

In the present study, we examined the mediation effect of different personality traits on the relationship between short-term perceived stress and total/crystallized cognition and explored the potential mechanism based on neuroimaging data. The relationship between stress and cognition was mediated by neuroticism, conscientiousness, and agreeableness. The mediation effect of neuroticism and agreeableness modulated the adverse effect of short-term perceived stress on cognition, whereas conscientiousness mitigated this adverse effect. The total effect, which was a combination of the direct effect of stress and indirect effect of personality, indicated that stress was negatively associated with total/crystallized cognition. Further neuroimaging analysis showed that right SFG cortical thickness was not only positively associated with stress and neuroticism, but also was negatively associated with total and crystallized cognition. The chain mediation model found that the right SFG and neuroticism play a small chain mediating effect between stress and total cognition. Moreover, people with high conscientiousness had a worse cognitive function and exhibited the hypoactivation of FC between the left lateral OFC and left superior medial frontal cortex. Our study indicates that people with high neuroticism have a higher vulnerability to cognitive decline induced by stress, while people with low neuroticism have a lower vulnerability to cognitive decline induced by stress. These results broaden the understanding of the relationship between short-term perceived stress, cognition, and neuroticism, and indicate that the structural and functional changes of SFG may be underlying neural mechanisms.

Short-term perceived stress was negatively associated with total/crystallized cognitive function, and the relationship was mainly mediated by neuroticism and conscientiousness, and partially mediated by agreeableness. Acute stress exposure and chronic perceived stress across the life span increase the neuroticism level [15, 59]. Individuals who score high on neuroticism are more likely than average to be moody and to experience negative feelings [60], respond worse to stressors, and are more likely to interpret ordinary situations as threatening and minor frustrations as hopelessly difficult. Continuous stress reduce conscientiousness which is associated with decreasing self-control and deliberation [16, 61], and then lower conscientiousness improved cognitive flexibility [29, 62, 63], and language comprehension [64]. Thus, decreasing conscientiousness was not completely harmful, and may have a positive indirect effect, contrary to the direction of the total effect on total cognition and crystallized cognition. This opposite direction could be interpreted in such a way that the modulatory effect of conscientiousness partly relieves the effect of stress on cognition, whereas neuroticism and agreeableness transmit the effect of stress on cognition. In the present study, the mediated effect of personality traits on crystallized cognition and not fluid cognition, maybe because personality traits have a higher correlation with crystallized cognition rather than fluid cognition [33, 65]. Support for the finding was provided by a study by Ashton and colleagues, which have reported openness is more strongly correlated with crystallized than with fluid abilities [66]. All in all, cognitive decline is caused by a combination of the direct effect of stress and the indirect effect of personality.

The modulatory effects of neuroticism and conscientiousness on this relationship were supported by the neuroimaging data. Firstly, the neuroimaging analysis found that right SFG cortical thickness was positively associated with perceived stress and neuroticism, and negatively associated with total/crystallized cognitive function. In addition, the chain mediation model further demonstrated that the cortical thickness of the right SFG and neuroticism play a small but significant chain mediating effect between perceived stress and total/crystallized cognition. The SFG, comprising one-third of the prefrontal cortex, contributes to higher cognitive functions and particularly to working memory and executive cognition [67]. The PFC is sensitive to the detrimental effects of stress exposure and the cortical thickness of PFC is associated with cognition impairement [34,68–70]. High levels of stress induced decreased blood flow and lower coherence regional homogeneity in the SFG [71, 72]. A recent study based on HCP also found that the association between the SFG thickness and aggressive behavior is mediated by neuroticism, and the SFG thickness is positively associated with neuroticism, which supports our results [73]. People with high neuroticism who perceive more insecurity show greater activation in right SFG [74] which mediates the processing of subjective awareness and self-related information [75]. The findings showed that individuals with a higher right SFG cortical thickness had higher neuroticism and worse total/crystallized cognitive function than individuals with lower right SFG cortical thickness. Secondly, we also found that the FC between the left lateral OFC and left superior medial frontal cortex is negatively correlated with conscientiousness and positively correlated with crystallized cognition. The OFC in the ventral surface of the prefrontal lobe is involved in the cognitive process of sensory integration, emotion processing, and decision-making [76–78]. And the superior medial frontal cortex which is part of SFG is correlated with inhibitory control ability and other cognitive function [67, 79]. The FC between left lateral OFC and left superior medial frontal cortex, the major component of the executive control network [80], is involved in cognitive control and decision-making [81]. Crystallized cognition refers to learned knowledge and experiences which enable people to categorize situations to make decisions [82], and people with high conscientiousness have poor decision-making capability due to the traits reflecting order and deliberation [63] which may explain why people with high conscientiousness have worse crystallized cognition than people with low conscientiousness.

The present study has several limitations. Firstly, the perceived stress in the study was measured by short-term perceived stress scales which assesses the degree to which situations are appraised as stressful in the last month and does not reflect the long-term stress level. Secondly, our study was based on whole-brain analysis without pre-defined regions of interest, and the further comparisons of results did not find overlapping FC in the AAL atlas. Therefore, the FC results are scattered and in-depth data analysis using another parcellation atlas is required to confirm the robustness of the current findings. Thirdly, the HCP data were cross-sectional without longitudinal follow-up, so it is difficult to prove a causal relationship between short-term perceived stress and cognitive function. Future studies should explore the influence of longitudinal changes in chronic stress and acute stress on cognitive function and the involvement of specific brain structure and neurocircuitry.

## Conclusion

The present study tested the hypothesis that short-term perceived stress is negatively associated with total/crystallized cognition, mediated by neuroticism, conscientiousness, and agreeableness. Our findings might be useful for understanding the neuroimaging mechanisms that underlie stress-related cognition decline and preventing and controlling the adverse effects of perceived stress among individuals with susceptible personality traits.

## Supplementary information

Supplementary Materials
