# Supplementary material for: The effect of perceived stress on cognition is mediated by personality and the underlying neural mechanism
Source: Transl Psychiatry. 2022 May 12;12:199. doi: 10.1038/s41398-022-01929-7 (PMC9098451; doi:10.1038/s41398-022-01929-7)
Supplement: Supplementary file 1 — Supplementary Materials [file 41398_2022_1929_MOESM1_ESM.docx]

**Supplementary Materials**

**Method**

**Cognitive function from the Human Connectome Project**

Cognition scores from the NIH Toolbox included total cognitive function composite score (including all toolbox cognition measures, comprising both fluid and crystallized cognition measures), crystallized cognition composite score (i.e., the ability to solve problems based on prior knowledge^1^ and experience, including language decoding and language comprehension), and fluid cognition composite score (i.e., the ability to solve novel reasoning problems^1^, which correlates with executive function, episodic memory, working memory, and processing speed) ^2^. The specific test mainly includes the following five items:

1. Language decoding and language comprehension are assessed using the NIH Toolbox oral reading recognition test and NIH Toolbox picture vocabulary test. The oral reading recognition test and picture vocabulary test are in a computer-adaptive test format that measures reading decoding skills and crystallized abilities. These abilities are generally more dependent on past learning experiences and consistent across the lifespan for ages 7-85.
2. Executive function is measured by two tests in the NIH Toolbox battery. Cognitive flexibility is assessed using the NIH Toolbox dimensional change card sort test (DCCS), and attention and inhibitory control are assessed using the NIH Toolbox flanker inhibitory control and attention test. The DCCS is considered a “fluid ability” measure, with performance generally increasing through childhood and then declining across the adult lifespan. The flanker inhibitory control and attention test is considered a fluid ability measure (i.e., it measures the capacity for new learning and information processing in novel situations), in which performance reaches a peak in early adulthood and then tends to decline across the lifespan.
3. Episodic memory is assessed using the NIH Toolbox Picture Sequence Memory Test. The Picture Sequence Memory Test involves the acquisition, storage, and effortful recall of new information. It is considered a strong “fluid ability” measure, with performance reaching a peak in early adulthood and declining across the lifespan. It involves recalling increasingly lengthy series of illustrated objects and activities that are presented in a particular order on the computer screen.
4. Working memory is measured using the NIH Toolbox List Sorting Working Memory Test. This task assesses working memory and requires the participant to sequence different visually and orally presented stimuli (foods and animals) into size order. List sorting is a measure of working memory, tapping both information processing and storage. It is considered a “fluid ability” measure, with performance tending to peak in early adulthood and then declining across the lifespan.
5. The speed of mental processing is measured using the NIH Toolbox pattern comparison processing speed test. The pattern comparison processing test is a measure of processing speed, which is considered a “fluid ability” because it steadily improves (i.e., the time to complete the task decreases) throughout childhood and adolescence and then begins to decline in adulthood.

**Construction of the whole-brain functional network**

After preprocessing, the whole brain (gray matter) was parcellated into regions of interest (ROI) using the AAL and Shen atlas. Then the time series were extracted in each ROI by averaging the signals of all voxels within that region. The parcellation of the brain used in this investigation was the Shen atlas^3^, which was found to be useful in this investigation because it has more areas than the AAL atlas. Brain regions based on the AAL atlas was bigger than Shen atlas so that the functional connectivity was more likely to be insignificant after averaging the regional blood oxygen level-dependent signals of all voxels within each region.

**
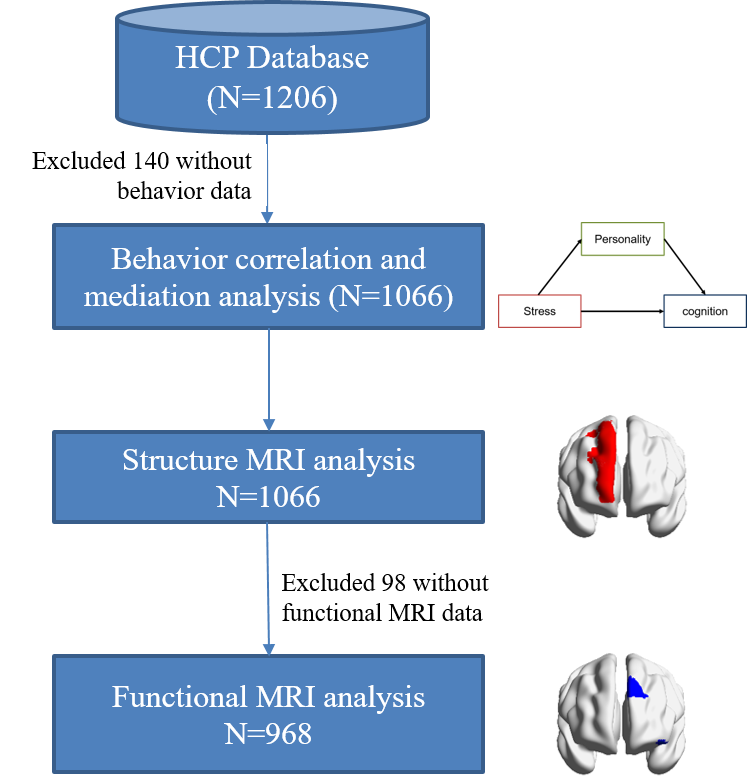
Figure S1. Flowchart of analyses.**

The analyses that were performed in this study, which were based on data from the Human Connectome Project (HCP), included behavior correlation and mediation analysis, shared brain structures, and functional connectivity based on MRI data. N, sample size for the analysis.

**Figure S2 Functional connectivity associated with total cognition, perceived stress, and personality traits using Shen atlas (n=968).**

a. Functional connectivity of total cognition included 3 edges and 6 nodes, which included the right temporal cortex, right temporal pole, right insula cortex, right lingual cortex, and right middle orbitofrontal cortex. b. Functional connectivity of perceived stress included 3 edges and 5 nodes, which included right parahippocampal cortex, right putamen nucleus, right middle occipital gyrus, left inferior temporal gyrus, and left inferior frontal gyrus. c. Functional connectivity of neuroticism included 11 edges and 18 nodes, which included bilateral frontal gyrus, bilateral precentral gyrus, right postcentral gyrus, left temporal pole, and left hippocampus cortex. d. Functional connectivity of openness included 2 edges and 4 nodes, which included right inferior frontal gyrus, left lateral orbitofrontal cortex, and left superior middle frontal gyrus. e. Functional connectivity of agreeableness included 1 edge and 2 nodes, which included right anterior orbitofrontal cortex and left lingual cortex. f. Functional connectivity of extroversion included 18 edges and 28 nodes, which included bilateral temporal cortex, bilateral fusiform cortex, bilateral lingual cortex, right medial frontal gyrus, right cingulate cortex, right cuneus nucleus, left superior marginal gyrus, left postcentral gyrus, left precentral gyrus, left precuneus gyrus, and left supplementary motor cortex.

**
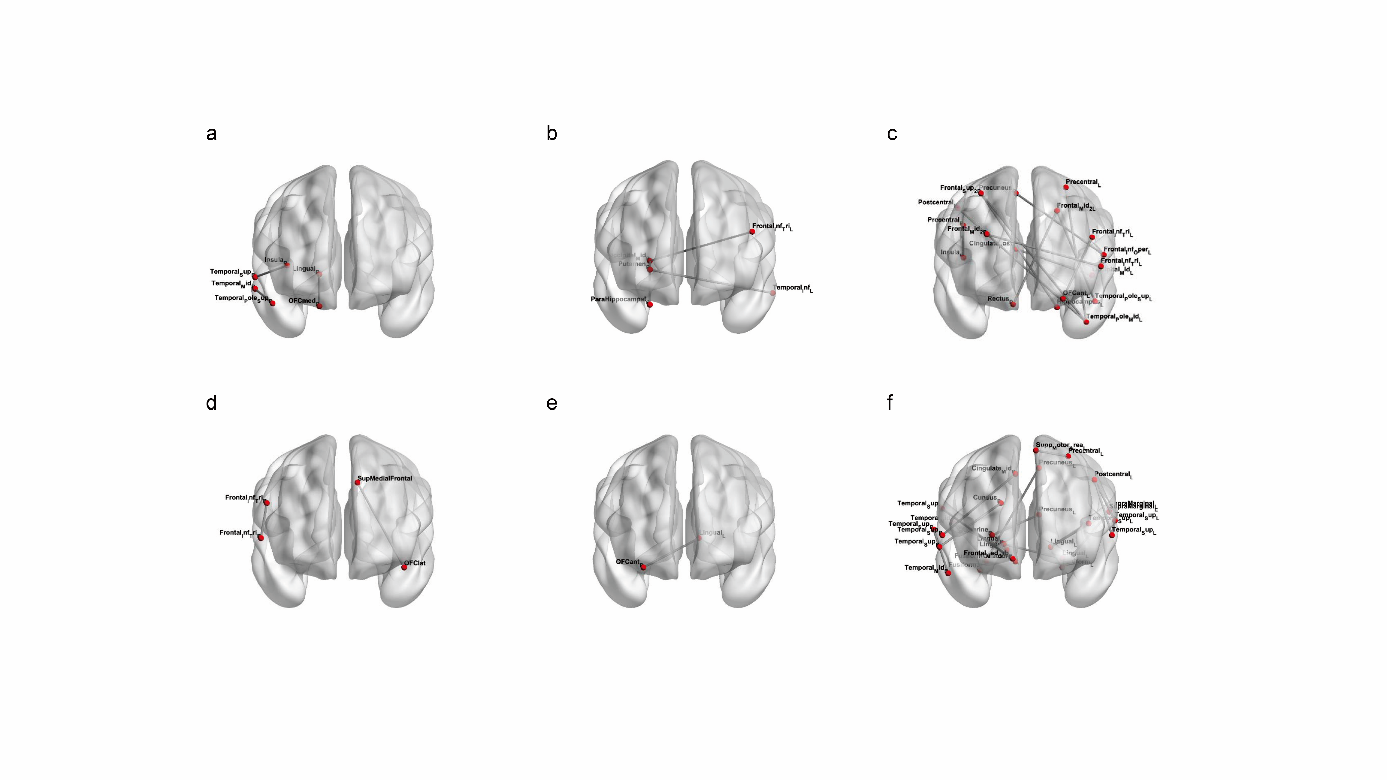
**

**Figure S3 Functional connectivity associated with total/crystallized cognition, perceived stress, and personality traits using AAL atlas (n=968).**

Except for openness, total/crystallized cognition, perceived, and personality traits have significant FC respectively. a. Functional connectivity of total cognition included 1 edge and 2 nodes, which included left caudate nucleus and left middle cingulum cortex. b. Functional connectivity of crystallized cognition included 1 edge and 2 nodes, which included right caudate nucleus and right angular gyrus. c. Functional connectivity of perceived stress included 1 edge and 2 nodes, which included right parahippocampal cortex and left hippocampus cortex. d. Functional connectivity of neuroticism included 4 edges and 6 nodes, which included right superior parietal gyrus, right postcentral gyrus, right superior middle frontal gyrus, left temporal pole, and left anterior cingulum cortex. e. Functional connectivity of conscientiousness included 2 edges and 4 nodes, which included right temporal pole, right amygdala cortex, right putamen, and anterior cingulum cortex. f. Functional connectivity of agreeableness included 1 edge and 2 nodes, which included right precentral gyrus and left supplementary motor cortex. g. Functional connectivity of extroversion included 4 edges and 7 nodes, which included right parahippocampal cortex, right cerebellum cortex, left medial frontal gyrus, left inferior orbital frontal gyrus, left superior marginal gyrus, and left superior parietal gyrus

**
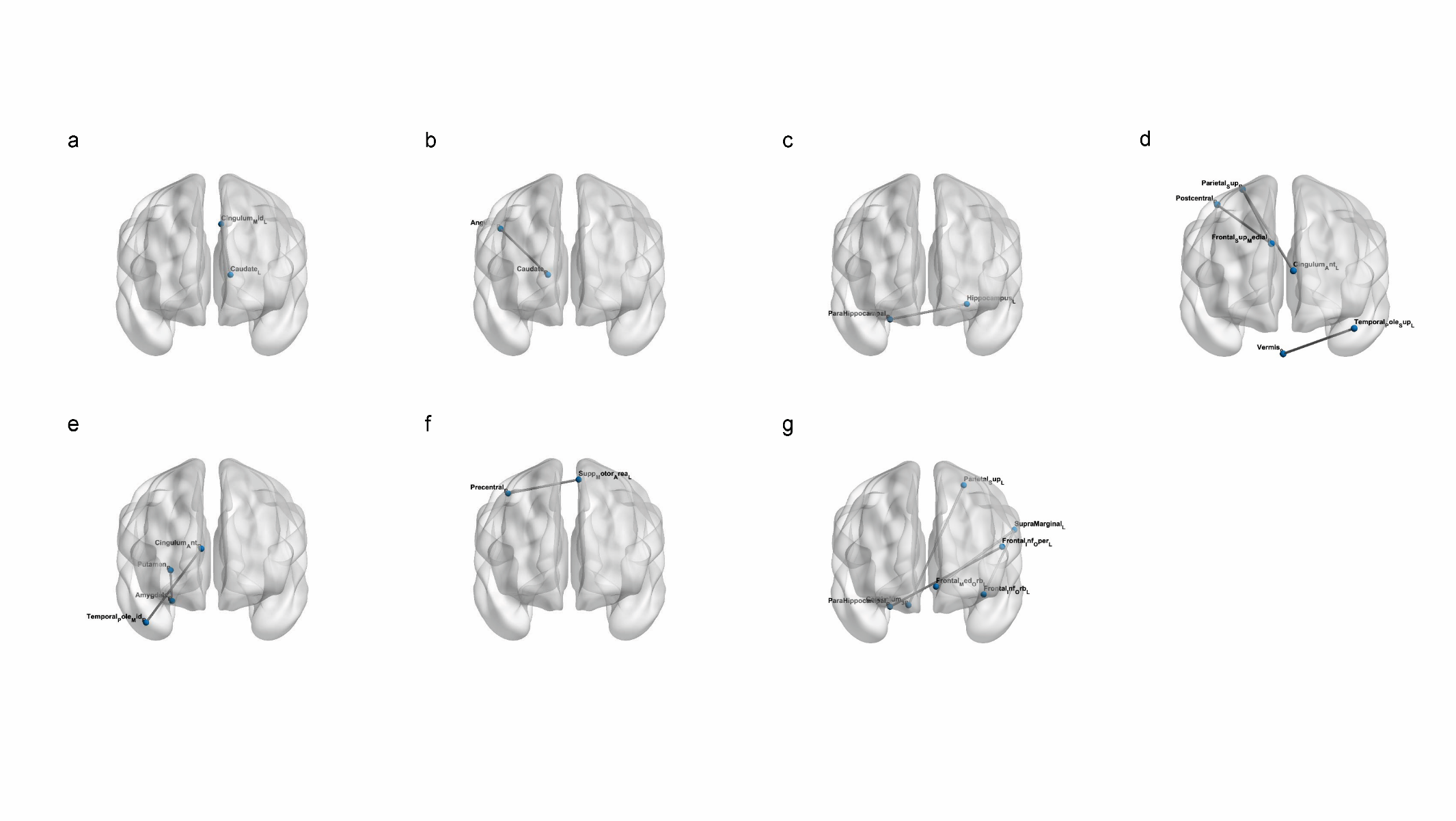
**

**Table S1. Simple mediation analysis of personality traits on relationship between perceived stress and cognition.**

| **X** | **M** | **Y** | **Indirect effect of X on Y** | | | | **Direct effect of X on Y** | | | | |
| --- | --- | --- | --- | --- | --- | --- | --- | --- | --- | --- | --- |
|  |  |  | *Effect* | *SE* | *LLCI* | *ULCI* | *Effect* | *SE* | *p* | *LLCI* | *ULCI* |
| Perceived stress | Neuroticism | **Total cognition** | -0.0504 | 0.025 | -0.1019 | -0.0035 | -0.0976 | 0.0602 | 0.105 | -0.2157 | 0.0204 |
|  |  | **Crystallized cognition** | -0.0311 | 0.0166 | -0.0652 | -0.0002 | -0.031 | 0.0392 | 0.4303 | -0.108 | 0.046 |
|  |  | Fluid cognition | -0.0297 | 0.0215 | -0.0732 | 0.0115 | -0.079 | 0.052 | 0.1291 | -0.1811 | 0.0231 |
|  | Openness | **Total cognition** | -0.0224 | 0.0124 | -0.0494 | -0.0003 | -0.1257 | 0.054 | 0.0201 | -0.2316 | -0.0197 |
|  |  | Crystallized cognition | -0.0225 | 0.0121 | -0.0471 | 0.0004 | -0.0396 | 0.0341 | 0.2467 | -0.1065 | 0.0274 |
|  |  | Fluid cognition | -0.0057 | 0.0046 | -0.0189 | 0.0001 | -0.103 | 0.0476 | 0.0305 | -0.1964 | -0.0097 |
|  | Conscientiousness | **Total cognition** | 0.0434 | 0.0126 | 0.0218 | 0.0715 | -0.1914 | 0.0556 | 0.0006 | -0.3005 | -0.0824 |
|  |  | **Crystallized cognition** | 0.039 | 0.0093 | 0.023 | 0.0604 | -0.1011 | 0.0359 | 0.005 | -0.1716 | -0.0305 |
|  |  | Fluid cognition | 0.014 | 0.0093 | -0.0022 | 0.0349 | -0.1227 | 0.0483 | 0.0113 | -0.2176 | -0.0279 |
|  | Agreeableness | Total cognition | -0.001 | 0.0095 | -0.0199 | 0.0181 | -0.1471 | 0.0558 | 0.0085 | -0.2566 | -0.0375 |
|  |  | **Crystallized cognition** | -0.0121 | 0.0064 | -0.0266 | -0.0011 | -0.0499 | 0.0363 | 0.1695 | -0.1212 | 0.0213 |
|  |  | Fluid cognition | 0.0083 | 0.0084 | -0.0069 | 0.0263 | -0.1171 | 0.0482 | 0.0153 | -0.2116 | -0.0225 |
|  | Extroversion | Total cognition | 0.0041 | 0.0043 | -0.0014 | 0.0169 | -0.1521 | 0.055 | 0.0058 | -0.2601 | -0.0442 |
|  |  | Crystallized cognition | 0.0047 | 0.0043 | -0.0024 | 0.0154 | -0.0668 | 0.0357 | 0.0618 | -0.1368 | 0.0033 |
|  |  | Fluid cognition | 0.0001 | 0.0023 | -0.004 | 0.0061 | -0.1089 | 0.0476 | 0.0224 | -0.2022 | -0.0155 |

X, input variable; M, mediation variable; Y, output variable; SE, Standard Error; LLCI, 95% of lower confidence interval; ULCI, 95% of upper confidence interval. If the CI does not include 0, then the indirect mediation effect is significant. The bold font means statistically significant.

**Table S2. Cortical area of brain regions associated with total, crystallized, or fluid cognition (n=1,066).**

| Brain region | **Total cognition** | | **Crystallized cognition** | | **Fluid cognition** | |
| --- | --- | --- | --- | --- | --- | --- |
|  | coefficient | *pval_fdr* | coefficient | *pval_fdr* | coefficient | *pval_fdr* |
| Left bankssts cortex | 0.3959 | 0.3647 | 0.6174 | 0.0334 | -0.0414 | 0.9240 |
| Left caudal anterior cingulate cortex | 0.9842 | 0.0222 | 0.5540 | 0.0435 | 0.6471 | 0.1165 |
| Left caudal middle frontal cortex | 1.3649 | 0.0030 | 1.0943 | 0.0004 | 0.5768 | 0.1574 |
| Left cuneus cortex | 1.9598 | 0.0001 | 0.6526 | 0.0297 | 1.6537 | 0.0007 |
| Left entorhinal cortex | 1.6862 | 0.0005 | 0.9201 | 0.0026 | 1.1641 | 0.0138 |
| Left fusiform cortex | 1.4279 | 0.0035 | 1.0656 | 0.0009 | 0.7285 | 0.1165 |
| Left inferior parietal cortex | 1.3609 | 0.0045 | 1.0959 | 0.0006 | 0.6516 | 0.1304 |
| Left inferior temporal cortex | 1.6928 | 0.0008 | 1.2726 | 0.0002 | 0.8049 | 0.0892 |
| Left insula cortex | 1.6147 | 0.0009 | 1.0869 | 0.0006 | 0.9431 | 0.0420 |
| Left lateral occipital cortex | 1.9226 | 0.0002 | 0.9836 | 0.0021 | 1.3447 | 0.0063 |
| Left lateral orbitofrontal cortex | 1.9547 | 0.0002 | 1.1728 | 0.0004 | 1.3080 | 0.0077 |
| Left medial orbitofrontal cortex | 0.8814 | 0.0505 | 1.0396 | 0.0006 | 0.1026 | 0.8194 |
| Left middle temporal cortex | 1.7865 | 0.0004 | 1.3321 | 0.0001 | 0.9341 | 0.0510 |
| Left paracentral cortex | 1.7160 | 0.0002 | 1.0521 | 0.0005 | 1.1148 | 0.0138 |
| Left pars orbitalis cortex | 1.0249 | 0.0272 | 0.6950 | 0.0226 | 0.5588 | 0.1843 |
| Left pars triangularis cortex | 0.8623 | 0.0515 | 0.1807 | 0.5240 | 0.9501 | 0.0345 |
| Left pericalcarine cortex | 1.8315 | 0.0002 | 0.6609 | 0.0226 | 1.5337 | 0.0007 |
| Left postcentral cortex | 1.7119 | 0.0006 | 1.4317 | 0.0000 | 0.7113 | 0.1165 |
| Left posterior cingulate cortex | 1.5250 | 0.0010 | 0.8709 | 0.0034 | 1.0077 | 0.0268 |
| Left precentral cortex | 2.0013 | 0.0002 | 1.2875 | 0.0001 | 1.1281 | 0.0217 |
| Left precuneus cortex | 1.8168 | 0.0003 | 1.2310 | 0.0003 | 1.0292 | 0.0345 |
| Left rostral anterior cingulate cortex | 1.4058 | 0.0030 | 1.0865 | 0.0005 | 0.6668 | 0.1205 |
| Left rostral middle frontal cortex | 1.8425 | 0.0003 | 1.5506 | 0.0000 | 0.7322 | 0.1165 |
| Left superior frontal cortex | 1.7620 | 0.0006 | 1.1806 | 0.0005 | 1.0588 | 0.0345 |
| Left superior parietal cortex | 1.5272 | 0.0014 | 0.9640 | 0.0017 | 0.9073 | 0.0487 |
| Left superior temporal cortex | 1.1073 | 0.0228 | 1.0161 | 0.0017 | 0.3725 | 0.4220 |
| Left supramarginal cortex | 1.8308 | 0.0003 | 0.9049 | 0.0046 | 1.3835 | 0.0059 |
| Left temporal pole cortex | 1.2478 | 0.0059 | 0.7511 | 0.0103 | 0.8330 | 0.0548 |
| Left transverse temporal cortex | 0.7680 | 0.0749 | 0.5990 | 0.0346 | 0.3340 | 0.4220 |
| Right bankssts cortex | 0.6250 | 0.1514 | 0.8278 | 0.0046 | 0.0518 | 0.9141 |
| Right caudal anterior cingulate cortex | 0.8285 | 0.0479 | 0.5439 | 0.0435 | 0.6191 | 0.1165 |
| Right caudal middle frontal cortex | 1.3259 | 0.0035 | 1.0439 | 0.0005 | 0.6354 | 0.1216 |
| Right cuneus cortex | 1.8697 | 0.0002 | 0.7598 | 0.0108 | 1.4893 | 0.0011 |
| Right entorhinal cortex | 1.2414 | 0.0058 | 0.8469 | 0.0037 | 0.6567 | 0.1165 |
| Right frontal pole cortex | 0.9993 | 0.0253 | 0.9789 | 0.0010 | 0.1984 | 0.6360 |
| Right fusiform cortex | 1.2809 | 0.0094 | 1.2073 | 0.0004 | 0.3181 | 0.4853 |
| Right inferior parietal cortex | 1.5141 | 0.0028 | 0.9153 | 0.0046 | 0.9031 | 0.0548 |
| Right inferior temporal cortex | 1.9025 | 0.0002 | 1.1304 | 0.0005 | 1.1531 | 0.0173 |
| Right insula cortex | 1.6588 | 0.0008 | 1.1059 | 0.0006 | 0.9827 | 0.0385 |
| Right isthmuscingulate cortex | 1.1359 | 0.0118 | 0.6567 | 0.0250 | 0.6682 | 0.1165 |
| Right lateraloccipital cortex | 1.4598 | 0.0029 | 0.6600 | 0.0333 | 1.1851 | 0.0150 |
| Right lateral orbitofrontal cortex | 1.6225 | 0.0010 | 1.0594 | 0.0009 | 0.9848 | 0.0381 |
| Right lingual cortex | 1.5610 | 0.0010 | 0.7137 | 0.0179 | 1.1180 | 0.0169 |
| Right medial orbitofrontal cortex | 0.8737 | 0.0565 | 0.6247 | 0.0376 | 0.4555 | 0.2891 |
| Right middle temporal cortex | 2.1229 | 0.0001 | 1.6668 | 0.0000 | 1.0090 | 0.0381 |
| Right paracentral cortex | 1.1097 | 0.0124 | 0.7605 | 0.0087 | 0.6492 | 0.1165 |
| Right parahippocampal cortex | 1.0678 | 0.0192 | 0.9497 | 0.0017 | 0.3240 | 0.4515 |
| Right pars opercularis cortex | 1.2355 | 0.0060 | 0.8499 | 0.0037 | 0.6653 | 0.1165 |
| Right pars orbitalis cortex | 1.3130 | 0.0053 | 0.6733 | 0.0257 | 0.8802 | 0.0536 |
| Right pars triangularis cortex | 0.8663 | 0.0479 | 0.4500 | 0.1086 | 0.6521 | 0.1165 |
| Right pericalcarine cortex | 1.7277 | 0.0002 | 0.5729 | 0.0435 | 1.4644 | 0.0011 |
| Right postcentral cortex | 2.0172 | 0.0001 | 1.3596 | 0.0000 | 1.0964 | 0.0221 |
| Right posterior cingulate cortex | 1.4622 | 0.0014 | 0.9957 | 0.0009 | 0.8419 | 0.0542 |
| Right precentral cortex | 2.3893 | 0.0000 | 1.5524 | 0.0000 | 1.4231 | 0.0053 |
| Right precuneus cortex | 1.4019 | 0.0038 | 0.8541 | 0.0062 | 0.8752 | 0.0548 |
| Right rostral anterior cingulate cortex | 0.9783 | 0.0275 | 0.7770 | 0.0080 | 0.5913 | 0.1482 |
| Right rostral middle frontal cortex | 1.3411 | 0.0061 | 1.4401 | 0.0000 | 0.3188 | 0.4853 |
| Right superior frontal cortex | 1.7868 | 0.0006 | 1.2182 | 0.0004 | 0.9213 | 0.0548 |
| Right superior parietal cortex | 1.1326 | 0.0183 | 1.1320 | 0.0005 | 0.2059 | 0.6367 |
| Right superior temporal cortex | 1.2034 | 0.0118 | 1.0537 | 0.0009 | 0.6112 | 0.1574 |
| Right supramarginal cortex | 1.2269 | 0.0093 | 0.9253 | 0.0027 | 0.6651 | 0.1216 |

*pval_fdr*, False Discovery Rate (FDR)-corrected *p* value, df=1052, adjusted by age, gender, race, handedness, years of education, total household income, body mass index (BMI), adult self-report (ASR) anxious/depressed (scale I) raw score, alcohol dependence, tobacco dependence diagnosis, marijuana dependence diagnosis, all positive test for drug, total white matter volume, and total gray matter volume.

**References**

1 Cattell, R. B. Theory of fluid and crystallized intelligence: A critical experiment. *J. Educ. Psychol.* (1963).

2 Akshoomoff, N., Beaumont, J. L., Bauer, P. J., Dikmen, S. S., Gershon, R. C., Mungas, D. *et al.* VIII. NIH Toolbox Cognition Battery (CB): composite scores of crystallized, fluid, and overall cognition. *Monogr* *Soc. Res. Child. Dev* **78**, 119-132, doi:10.1111/mono.12038 (2013).

3 Shen, X., Tokoglu, F., Papademetris, X. & Constable, R. T. Groupwise whole-brain parcellation from resting-state fMRI data for network node identification. *Neuroimage* **82**, 403-415, doi:10.1016/j.neuroimage.2013.05.081 (2013).
